# The influence of motor tasks and cut-off parameter selection on artifact subspace reconstruction in EEG recordings

**DOI:** 10.1007/s11517-020-02252-3

**Published:** 2020-08-28

**Authors:** Phillipp Anders, Helen Müller, Nina Skjæret-Maroni, Beatrix Vereijken, Jochen Baumeister

**Affiliations:** 1 Department of Neuromedicine and Movement Science, Norwegian University of Science and Technology (NTNU), Trondheim, Norway; 2 Department Exercise & Health, Paderborn University, Paderborn, Germany

**Keywords:** Artifact, Data processing, Electroencephalography

## Abstract

Advances in EEG filtering algorithms enable analysis of EEG recorded during motor tasks. Although methods such as artifact subspace reconstruction (ASR) can remove transient artifacts automatically, there is virtually no knowledge about how the vigor of bodily movements affects ASRs performance and optimal cut-off parameter selection process. We compared the ratios of removed and reconstructed EEG recorded during a cognitive task, single-leg stance, and fast walking using ASR with 10 cut-off parameters versus visual inspection. Furthermore, we used the repeatability and dipolarity of independent components to assess their quality and an automatic classification tool to assess the number of brain-related independent components. The cut-off parameter equivalent to the ratio of EEG removed in manual cleaning was strictest for the walking task. The quality index of independent components, calculated using RELICA, reached a maximum plateau for cut-off parameters of 10 and higher across all tasks while dipolarity was largely unaffected. The number of independent components within each task remained constant, regardless of the cut-off parameter used. Surprisingly, ASR performed better in motor tasks compared with non-movement tasks. The quality index seemed to be more sensitive to changes induced by ASR compared to dipolarity. There was no benefit of using cut-off parameters less than 10.

**Graphical abstract:**
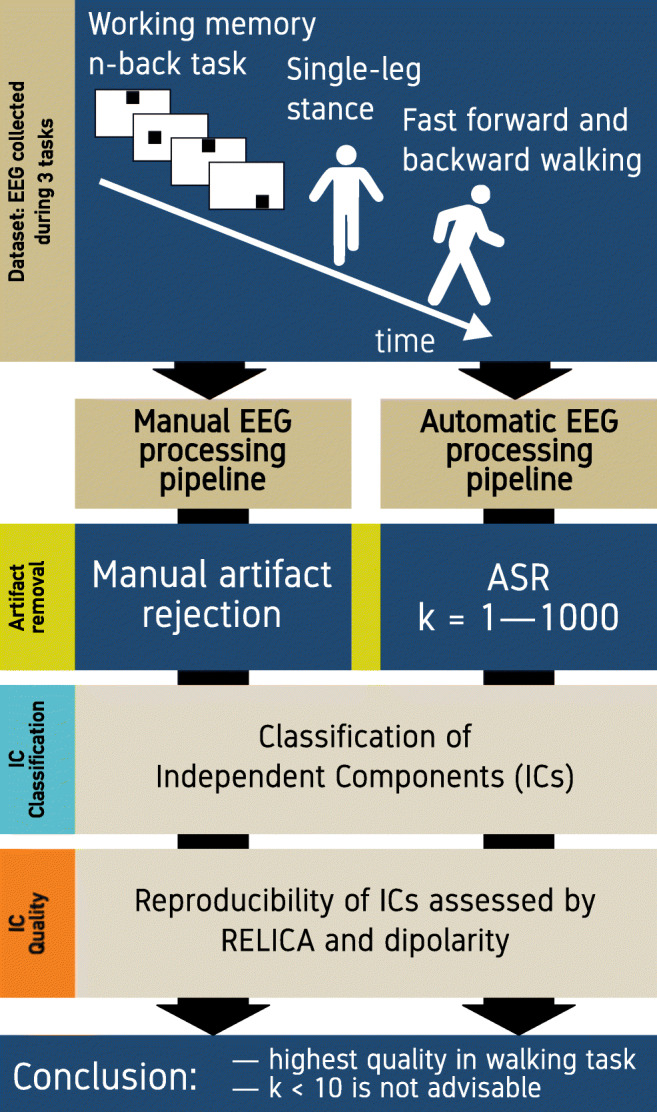
The graphical abstract shows the three tasks performed during EEG recording, the two processing pipelines (manual and artifact subspace reconstruction), and the metrics the conclusion is based on.

The graphical abstract shows the three tasks performed during EEG recording, the two processing pipelines (manual and artifact subspace reconstruction), and the metrics the conclusion is based on.

## Introduction

Electroencephalogram (EEG) is one of the most used methods to record activity of the brain in both clinical and applied research (e.g. epilepsy and exergaming, e.g. Acharya et al. [1] and Anders et al. [2]). Recent developments in both hardware and software, such as active electrodes [3] and advanced filter algorithms [4] make it possible to record usable EEG, while participants perform tasks involving physical movements or even in real-world environments. This offers neuroscientists a plethora of novel research designs, such as the concurrent measurement of brain activity during the execution of motor tasks, instead of having to rely on pre-post EEG comparisons or minimal participant behavior, thereby enabling the development of more natural behavior models [5].

However, the analysis of brain activity measured while participants perform motor tasks remains challenging due to the lower signal-to-noise ratio compared with, e.g., resting state analyses. EEG recordings are typically contaminated with non-brain related signals such as eye blink artifacts, artifacts due to impedance changes caused by a relative shift between the electrode and the skull, and artifacts due to electrical activity produced by facial and skeletal muscles. In general, the likelihood of the occurrence of the latter two types of artifacts increases with the vigor of the motor task.

A commonly applied strategy to isolate stereotypical noise sources such as eye blinks or repetitive motion artifacts is the use of independent component analysis (ICA) [6]. In ICA, an inverse model is used to reveal the independent components (ICs), that is, the sources of the cortical activity. ICs can then be classified as functional, non-functional, or a mixture of both. In order to create the inverse model used for the calculation of ICs, the EEG data needs to be cleaned, i.e., long-term signal non-stationarities [7] and large transient and non-repetitive artifacts [8] need to be removed.

Traditionally, EEG experts with experience in data cleaning remove transient artifacts and noise-contaminated channels through visual inspection [2, 9]. A major disadvantage of the current state-of-the-art manual artifact removal process is the loss of data. If an artifact is present in one channel, data from corresponding time series in all other channels has to be removed as well. This problem has become even more pronounced with the advent of high-density EEG systems (> 128 channels), since the likelihood for electrode shifts which cause the rejection of data increases with the number of EEG channels. Furthermore, manual cleaning of EEG data using visual inspection is not fully reproducible and time-consuming and requires extensive experience.

The recent advances in EEG processing algorithms [7] pave the way towards an automated and standardized method to remove artifacts. From a practical perspective, automatic pre-processing would speed up data processing as it would serve as a replacement, either in full or in part, of time-intensive manual artifact removal. This advantage becomes even more pronounced when EEG datasets are of long duration or are recorded using high-density EEG systems.

An important step towards automated and reproducible EEG preprocessing in research is the development of recent filter algorithms that originated in the field of brain-computer-interfaces (BCIs). One promising example is the artifact subspace reconstruction (ASR) [10]. ASR creates a robust covariance matrix based on the cleanest parts of an EEG recording. Subsequently, principal component analyses are performed in a sliding window of 1 second. A window is rejected if the standard deviation of a principal component exceeds the standard deviation of the automatically chosen cleanest part of the EEG recording multiplied by a tunable cut-off parameter (*k*). A rejected window is then reconstructed using the covariance matrix. A more detailed description of ASR can be found in [11–14].

The degree of reconstruction in ASR is influenced by the selected cut-off parameter *k*. However, selection of appropriate cut-off parameters is challenging as the literature background is sparse and studies typically underreport cut-off parameters used in their processing pipelines. A rare exception are the studies of Chang et al. [11, 14] on EEG data recorded while participants performed a simulated driving task. They found that cut-off parameters between 10 and 100 or 20–30, respectively, and delivered the best results in terms of eye artifact removal and conservation of brain activity. No studies are currently available in which participants performed more vigorous motor tasks during EEG recordings. It is therefore not known whether ASR can be used in such tasks and how they affect which ASR cut-off parameters are appropriate.

Following the identified gaps in our knowledge, the aim of the current study was to investigate how movement vigor and choice of cut-off parameters in ASR affect properties of the EEG data. To this end, we (1) assessed the ratio of EEG data removed or reconstructed in sensor-space for three tasks that required different amounts and vigor of movement when using an automated preprocessing pipeline including ASR using 10 different cut-off parameters, compared to manually cleaned EEG data using visual inspection, (2) evaluated the reproducibility and dipolarity of the resulting ICs, and (3) assessed whether either of these qualities were affected by the cut-off parameter or the task. Furthermore, we assessed the number and quality of functional ICs depending on the task and the cut-off parameter used.

## Methods

### Sample population

To assess the effect of task on the artifact removal performance of ASR, we used recorded EEG instead of simulated EEG in order to test the algorithms under conditions as close to reality as possible. A convenience sample of five healthy young participants (all female; age: 23.2 ± 2.58 years, height: 172.4 ± 3.13 cm, weight: 63.8 ± 4.38 kg) was recruited.

All procedures performed in this study were in accordance with the ethical standards of the institutional review board of the University of Paderborn and with the 1964 Helsinki declaration and its later amendments. All participants provided informed consent prior to data collection.

### Procedure

We recorded continuous EEG data during three tasks that required different amounts of movement. All participants performed the tasks in the same order.

The first task was a seated working memory *n*-back task, with 10 sets of 30 stimuli of 2 s each. The participants looked at a computer display presenting a 3 by 3 dot matrix. If the current pattern was the same as the pattern three pictures before, they had to press a button with their right thumb. If not, the participant had to press a button with their left thumb.

The second task consisted of 20 alternating single-leg stance phases held for 30 s each, with a break of 10 s between consecutive stance phases.

The third task consisted of two repetitions of a fast forward and backward walking task of 5.5 min each, using the Witty SEM (Microgate Slr, Bolzano, Italy). Five LED lamps were mounted on tripods and placed at 0°, ± 22.5°, and ± 45° from the participant’s point of view at a distance of 2.5 m. When one of the five LED lamps was switched on, the participants were asked to walk swiftly, not run, towards the lit LED lamp and to cover the light using their right hand before walking backwards to their starting position. This process was repeated until the end of the task. Both repetitions were combined into one EEG recording and treated as a single recording in further analyses.

### Data acquisition

Brain activity was recorded at 500 Hz using an EEG system consisting of a 64 channel Ag/AgCl active wet electrode elastic cap (Easycap, Herrsching, Germany) in an extended 10–20 electrode layout [15] and a wireless amplifier (Live Amp, Brain Products GmbH, Gilching, Germany) placed in a backpack to relief stress from the cables. The impedance was kept below 25 kΩ, in accordance with the manufacturer’s recommendations.

In order to ensure comparability of dipole locations between conditions, the electrode cap was not moved or manipulated between conditions. Furthermore, no gel was reapplied to the electrodes after participant preparation.

### Preprocessing

Data processing was performed in EEGLAB 14.1.1b [16], a toolbox for Matlab (Mathworks Inc., Nantick, MA).

In order to remove sinusoidal noise at 50 Hz and their harmonics, the CleanLine plug-in [7] was used. A band-pass filter with limiting frequencies of 3 and 30 Hz [17] was used to remove disturbances caused by both direct current drift and higher frequency disturbances such as electrical activity caused by the innervation of skeletal muscles.

After the removal of line noise and band limitation, all EEG data was copied to obtain 11 identical datasets, which were subsequently processed separately.

In one dataset, after re-referencing to average and downsampling to 250 Hz, an EEG expert removed noise contaminated channels and transient, non-stereotypical artifacts using visual inspection.

The remaining 10 datasets were preprocessed using the artifact subspace reconstruction [10] implemented in the clean_rawdata plug-in [12] separately for each task and participant. Channels were removed when poorly correlated (*r* < 0.85) to neighboring channels or when non-transient noise exceeded 4 SDs. ASR then reconstructed time windows contaminated with transient artifacts that exceeded *k* SDs based on the automatically chosen reference data or removed time windows when more than 25% of the remaining channels exceeded the threshold cut-off parameter. The cut-off parameter* k* was set to 1, 2, 5, 10, 20, 50, 100, 200, 500, and 1000, respectively. The cut-off parameters were chosen to cover the same range as in [11, 14]. Due to the expected computation time, we opted to use cut-off parameters that would result in equal intervals between values on a logarithmic scale. The available random-access memory for ASR was limited to 8 GB on a 16 GB computer to ensure equal availability for all iterations of automatic preprocessing. The 10 automatically processed datasets were subsequently re-referenced to average and downsampled to 250 Hz.

Subsequently, the following processing steps were applied to all 11 datasets in preparation for source space analysis:

Data of removed channels was interpolated using the EEGLAB function pop_interp in order to avoid bias towards a hemisphere with more remaining channels. This does not change the number of resulting ICs, as the rank of the matrix remains unchanged.

Spatiotemporal sources of brain activity were calculated by using an adaptive mixture independent component analysis (AMICA) [18, 19]. The locations of the spatiotemporal sources were determined by the dipfit plug-in for EEGLAB [20] based on a boundary element model [21, 22]. The fitTwoDipoles plug-in [23] was used to account for bilaterally symmetrical ICs.

ICs were classified into seven categories, namely “brain,” “muscle,” “eye,” “heart,” “line noise,” “channel noise,” and “other,” using the IClabel plug-in [24]. We chose this classification algorithm based on classification performance and computation time.

### Quality assessment of the results in source-space

In order to assess reproducibility of ICs across participants, we used the EEGLAB plug-in RELICA [25]. In RELICA, BeamICA [26], a less computationally expensive ICA compared with AMICA, allows for bootstrap statistics, which provides a quality index for all discovered ICs. BeamICA was set to use “point-by-point” mode with 50 iterations. The quality index is a measure for the dispersion of resulting ICs and can thereby be used to assess the reproducibility of ICs in terms of their localization. Subsequently, source localization as described above was applied to calculate the dipolarity of the ICs, as brain-related ICs are dipolar [8]. Dipolarity is a measure for how well the estimated dipole explains the original data. It describes the percentage to which a scalp map of an independent component can be explained by the scalp projection of a single equivalent dipole [4, 27]. The resulting dipolarity and quality index coordinates were used to classify the ICs into four categories (I, II, III, and the “forbidden region”), as described in [25]. ICs in category I are highly dipolar and reproducible (dipolarity > 0.85 and a quality index > 0.95). ICs in category II are a combination of brain signal and artifacts or a mixture of multiple cortical processes (dipolarity ≤ 0.85 and quality index > 0.95). ICs in category III are an inseparable mixture of artifacts and brain signals (quality index ≤ 0.95). ICs in the last category, the so-called “forbidden region,” have high dipolarity but low-quality index (dipolarity > 0.75 and quality index of < 0.45).

### Statistical analyses

Because the data was non-normally distributed, Kruskal-Wallis tests by ranks were used in R [28] to assess the effects of task and cut-off parameter on the quality indices and dipolarity, the number of ICs classified as brain related and the certainty of the classification as brain-related ICs. Wilcoxon’s signed-rank tests were used as follow-up in case of significance. The resulting *p*-values after the Wilcoxon’s signed-rank tests were corrected for multiple comparisons using Benjamini and Hochberg’s [29] method. The level for significance was set to *p* < 0.05.

## Results

Below, we first present the ratio of data removed and reconstructed for each task using ASR compared with the amount of data removed using visual inspection. Secondly, the dipolarity and reproducibility measured using the quality index calculated by RELICA for each task and cut-off parameter is presented. Thirdly, we present the classification results of ICs using IClabel.

### Ratio of data removed and reconstructed using ASR

As to be expected, lower cut-off parameters led to higher removal and reconstruction ratios. The walking task showed the highest overall removal ratio after visual inspection compared with single-leg stance task and the working memory task.

**Fig. 1:**
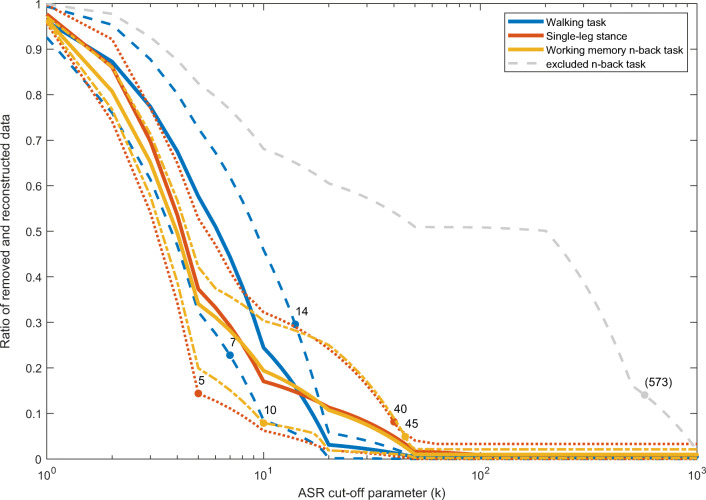
Mean ratio and range of removed and recovered data using ASR for cut-off parameters (*k*) between 1 and 1000 for each of the three tasks. The dots indicate the intersection of the ratio of removed data after manual cleaning using visual inspection of the preprocessed EEG for each participant. The dashed gray line represents EEG of a single participant during the *n*-back task

Figure 1 shows the ratio of removed and reconstructed EEG data in sensor-space for ASR cut-off parameters ranging from 1 to 1000 for each of the three tasks. Each line represents one participant performing one task. The dots indicate the intersection of each line with the respective ratio of manually removed data after visual inspection by an EEG expert. Linear interpolation was used to estimate the ratio of removed and reconstructed data in between calculated data points.

All curves showed a similar shape, except the curve of one participant performing the *n*-back task (dashed line). This was likely caused by a sudden onset of excessive noise in channel CP4 after 311 s of 600 s, resulting in larger ratios of data reconstructed. Excluding this task for this participant, the resulting ranges of cut-off parameters across participants were 7–14 for the walking task, 5–40 for the single-leg stance task, and 10–45 for the working memory *n*-back task.

The intersection of the ratio of manually removed data after visual inspection and the ratio curve of automatically preprocessed data using ASR for the single-leg stance task was at a cut-off parameter equal to 5 for one participant. For the other participants, the intersections in this task were located in the range between 20 and 40.

**Fig. 2:**
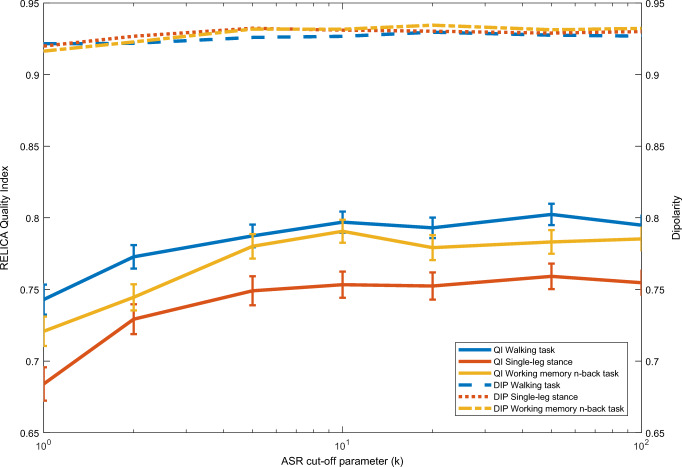
Resulting mean quality index and mean dipolarity of independent components for all tasks preprocessed using ASR as calculated by RELICA for cut-off parameters of 1–100 only, using ICs with a dipolarity greater than 0.85. The error bars indicate the standard error of the measurements

**Fig. 3:**
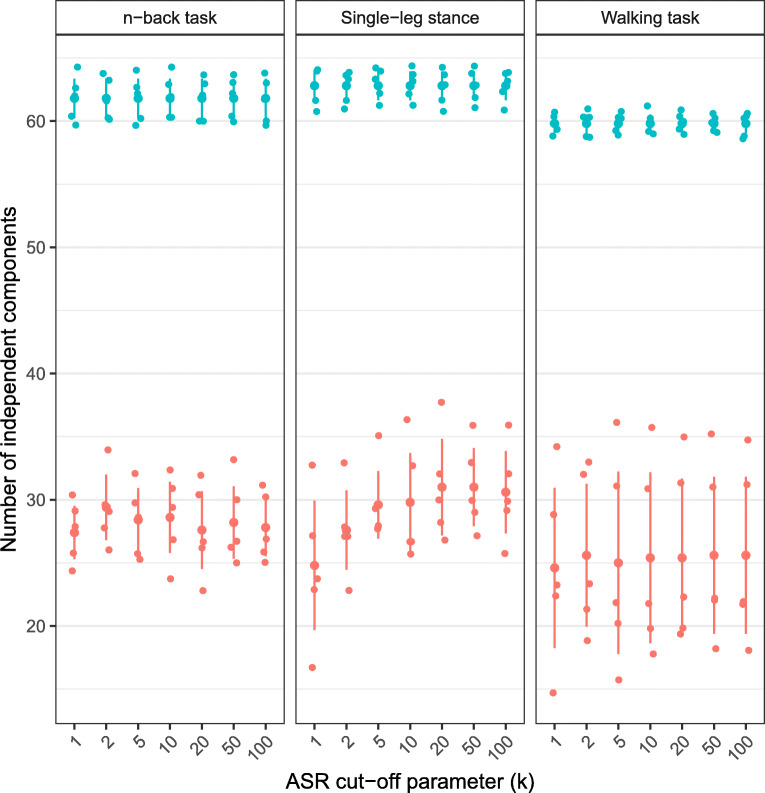
The number of ICs for each task identified by BeamICA. Teal dots represent all ICs, and red dots represent ICs with a dipolarity of > 0.85. The vertical bars indicate the standard error of the measurements

As indicated, ASR removes and reconstructs EEG data based on the input parameters. As can be seen in Fig. 1, no EEG data was reconstructed when using cut-off parameters larger than 100. The flat lines parallel to the x-axis for the range above 100 show the amount of data removed without being reconstructed by ASR. Based on this, we present results for cut-off parameters between 5 and 100 only in Figs. 2 and 3, in order to enhance readability.

### Dipolarity and quality of independent components

The quality indices calculated for each task and across all cut-off parameters using the RELICA plug-in for ICs with a dipolarity of > 0.85 increased until it reached a plateau at a cut-off parameter of 10 (solid lines in Fig.2). The total number of all ICs and the remaining ICs after removing ICs with a dipolarity of < 0.85 can be seen in Fig. 3. Quality index curves for all tasks showed roughly the same general behavior. The standard errors were comparable in size across all cut-off parameters and tasks. The highest quality indices were recorded for the walking task, followed by the *n*-back task, and the single-leg stance task. Dipolarity, on the other hand, only showed a slight increase towards higher cut-off parameters (dashed lines in Fig.2). The dipolarity and quality indices of the subset of ICs with a dipolarity of 0.85 or higher and preprocessed using ASR with cut-off parameters of one and two were significantly lower than the remaining ICs preprocessed with cut-off parameters of > 2 (all *p*’s < 0.001, except for *k* = 2 versus 5 and 10: *p* < 0.01 and *p* < 0.005, respectively). Furthermore, the dipolarity of ICs discovered in the fast-walking task was significantly lower (*p* < 0.05) compared with the ICs in both other tasks. Furthermore, the quality indices of ICs discovered in EEG recorded during the walking task were significantly higher than the quality indices of ICs of the remaining tasks (both *p*’s < 0.001, see Fig. 2). The resulting quality indices for ICs based on data processed using cut-off parameters of one or two were significantly lower compared with IC-based preprocessed using the remaining cut-off parameters (all *p*’s < 0.001, except for k = 2 versus 5: *p* < 0.005).

The majority of ICs were sorted in category III [25], indicating that there is inseparable noise mixed into the ICs. Only a few ICs were sorted as category I (both quality index and dipolarity above retention threshold) or category II (either artifact or a mix of two or more processes). We found 21 ICs in the “forbidden region,” where dipolarity is larger than 0.75 but the quality index below 0.45. As BeamICA did not deliver consistent results in any of the tasks for the manually cleaned data, resulting in quality indices of 0, these are not included in Fig. 2.

### Classification of independent components

**Fig. 4:**
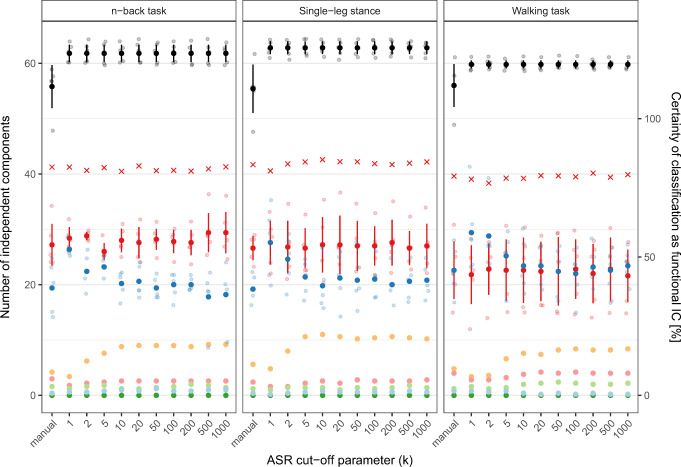
Independent components for EEG data preprocessed using ASR with cut-off parameters ranging from 1 to 1000 and manually cleaned EEG data. Spatiotemporal features were calculated using an adaptive mixture-independent component analysis. Total number of independent components (black) and independent components classified into seven categories using IClabel (red, “brain”; blue, “other noise”; other colors, “muscle,” “eye,” “heart,” “line noise,” and “channel noise”). The bars indicate the standard error. The crosses indicate the certainty of the classification of independent components as brain-related

Compared with manual cleaning, using ASR to clean the data resulted in more ICs due to fewer removed channels, as can be seen in Fig. 4 (black dots).

The automatic classification of ICs into functional or brain-related ICs and non-functional ICs revealed no difference in the number of brain-related ICs for either cleaning method (red dots). As expected, the number of ICs classified as brain activity by the IClabel plug-in was significantly lower in the walking task compared with the other conditions (both p’s < 0.001). There was no clear trend whether the cut-off parameter influenced the number of discovered brain-related ICs in this dataset, which was confirmed by the non-significant result of the Kruskal-Wallis test (χ^2^ = 1.7163, df = 10, *p* = 0.9981).

No ICs were classified as line noise since the CleanLine plug-in was used to remove line noise, as well as a bandpass filter with an upper edge frequency of 30 Hz.

IClabel classified more ICs as “other noise” when cut-off parameters of *k* = 1, 2, and 5 where used compared with higher ASR cut-off parameters or manual cleaning. This result seems to be due to fewer ICs being classified as “muscle” (orange dots). The number of ICs classified as “eye”-related (light green) seems to be largely unaffected by the cut-off parameter and cleaning method.

Furthermore, the certainty of the classification is based on the mean probability value that can be interpreted as a measure of classifier confidence in the discrete classification as brain-related IC [24, 30] seems to be unaffected by both the method of cleaning used and the cut-off parameter used in automatic cleaning (red crosses).

However, the certainty of the classification as brain-related IC was affected by the type of task, and significantly lower in the walking task than in the other two tasks (both p’s < 0.001). Furthermore, the classification certainty of brain-related ICs in the n-back task was significantly lower compared with the single-leg stance (*p* < 0.05).

## Discussion

The present study investigated the effect of ASR cut-off parameters on characteristics of EEG data recorded under three different tasks (*n*-back task, single-leg stance, and short bursts of fast walking). The effect of task and cut-off parameter was assessed using (1) the ratio of data removed or reconstructed using ASR in sensor-space, (2) the dipolarity and reproducibility of ICs as assessed by RELICA, and (3) the classification of ICs using IClabel. This knowledge is needed, particularly when motor tasks are employed with higher likelihood for causing movement artifacts such as in postural control or walking tasks.

ASR, as a preprocessing step before ICA, delivered the best results in terms of reproducibility and dipolarity in EEG recorded during bursts of fast walking compared with EEG recorded during a cognitive task and single-leg stance. The number and certainty of the classification as brain-related ICs were unaffected by the choice of cut-off parameter across all tasks. The cut-off parameters resulting in the same ratio of data removed and reconstructed as in manual cleaning using visual inspection revealed a lower range of equivalent cut-off parameters in the walking task compared with the cognitive and single-leg stance task.

### Ratio of data removed and reconstructed using ASR

Tasks more likely to cause movement artifacts seem to require lower cut-off parameters for ASR, when compared with the ratio of removed data during manual cleaning, as shown in Fig. 1. The ranges of cut-off parameters determined by the comparison to manually cleaned data for the single-leg stance task and the *n*-back task (Fig. 1) show rough agreement with recent literature. Mullen et al. [12] used cut-off parameters between 5 and 7. However, their application of ASR was in a BCI system using dry electrodes. Chang et al. [11] recommended to use cut-off parameters between 10 and 100 which was later adjusted to cut-off parameters between 20 and 30 [14]. These results were based on EEG recorded while performing a simulated driving task. Our results show that a human rater would remove a similar percentage of EEG as ASR with previously recommended cut-off parameters in the single-leg stance task and the *n*-back task. The range of cut-off parameters in the walking task was lower than the recommendations in recent literature. A possible explanation could be differences in the level of movement artifact contamination compared with the EEG used in [11, 14]. The walking task in our study might show higher levels of contamination as walking is a more vigorous task compared to simulated driving.

### Dipolarity and reproducibility of independent components

Surprisingly, significantly higher quality indices were observed for ICs in the walking task. This indicates the best reproducibility of ICs using EEG from this task. This finding was not anticipated since the walking task was the most contaminated with movement artifacts. The single-leg stance task showed the lowest quality indices, despite being the task with presumably the second lowest likelihood for causing artifacts. A possible explanation for this result might be that ASR partly removed brain-related activity in addition to removing noise and artifacts, since alpha waves were the most prominent feature in the EEG from this task.

The high plateau for all quality indices for cut-off parameters higher than 10 indicates that the quality of ICs is negatively affected by more aggressive cut-off parameters. This supports the findings of Chang et al. [14] that cut-off parameters of less than 20 are not advisable and resulted in significantly lower quality indices and dipolarity across tasks.

Interestingly, dipolarity was not affected as severely as the quality index by the choice of cut-off parameters. A consequence of this is that dipolarity alone might not be the optimal tool to determine whether an IC is brain-related and of high quality or not. Although computationally expensive, RELICA can potentially serve as a more dependable assessment tool for quality assurance of source-space results.

The overall quality of ICs was lower than those of Artoni et al. [25]. Our results are likely related to the amount of movement and the thereby induced artifacts. However, the cognitive task had no, or a very limited amount, movement. The reason for the difference in the quality index may be related to Artoni et al. using event-related potentials in their experiment whereas we used continuous EEG recordings.

Contrary to the findings of Artoni et al. [25], we found ICs in the “forbidden region.” They hypothesized that highly dipolar ICs cannot have extremely low-quality indices. Most ICs in the “forbidden region” (17 out of 21) were calculated using EEG preprocessed with ASR with a cut-off parameter of 10 or less. Twelve of those ICs used a cut-off parameter of 1. This indicates that low cut-off parameters may reduce the quality indices but do not affect the dipolarity of ICs. The single-leg stance task was the most prominent task in the “forbidden region,” with 15 of the 21 ICs. It seems possible that ASR, when used with cut-off parameters below the recommended range (k < 20) [14], can have a negative effect on the reproducibility of ICs. This assumption is supported by the significant difference between ICs preprocessed with cut-off parameters of one or two compared with the remaining cut-off parameters used.

Unfortunately, it was not possible to calculate quality indices of ICs that were based on manually cleaned data using RELICA. To further investigate whether this was related to eye blinks or remaining movement artifacts, we used the RELICA plug-in on manually cleaned and artifact-free seated, open-eyes baseline data from a previous study [6]. Despite this dataset not containing any movement contamination but only artifacts due to eyeblinks, we arrived at similar results. It was possible to obtain plausible resulting values from RELICA after removing the eyeblink artifacts from either EEG data set using ASR, ruling out the possibility that manual cleaning with resulting boundaries between the remaining EEG caused the issue. Although we cannot answer for sure what may have caused RELICA to perform unsatisfactory in manually cleaned EEG data, it seems reasonable to assume that the prominent eyeblink artifacts in both cases had an important role in its inability to deliver quality indices.

### Classification of independent components

The number of functional ICs classified by IClabel in each task remained constant regardless of the cut-off parameter used. This indicates that ASR does not lead to an artificial increase of brain-related ICs. Significantly more ICs were classified as functional in the n-back task and the single-leg stance task compared to the walking task. This might be due to the level of movement artifact contamination that remained in the EEG after cleaning. For the walking task, more ICs were needed to model the remaining noise, hence leaving fewer ICs available for functional processes.

The classification of noise sources was influenced by cut-off parameters less than 10 across all three tasks. Hence, it can be assumed that ASR, when used with cut-off values below 10, alters the noise patterns in the EEG, so that they cannot be distinguished by the automatic IC classification tool IClabel. This led to more ICs being classified as “other noise” instead of being distinguished into specific classes of noise. This effect was most notable in the class of “muscle” ICs. Therefore, it seems reasonable to assume that strong high-frequency broadband activity used to classify ICs as “muscle”-related was affected by the reconstruction of the signal by ASR [24]. The number of functional ICs remained constant, suggesting that ASR preserves functional ICs across cut-off parameters. Further research is needed to further support this interpretation.

### Next steps

Manual cleaning is likely to remove EEG linked to high intensity movements, whereas ASR reconstructs them. A potential implication of this is a bias either due to systematic removal of EEG or due to the reconstruction of EEG. Especially in mobile brain/body imaging applications [5], EEG recorded during movements is of high interest and an important topic for future research. There are still many unanswered questions such as which parameters can be used to automate the selection of cut-off parameters used in ASR and whether there is a bias introduced by the reconstruction of data.

Our results based on two motor tasks and one cognitive task indicate that it may not be possible to provide general recommendations for the choice of ASR cut-off parameters across different types of tasks performed while measuring brain activity. However, detecting the plateau in quality indices calculated using RELICA might be a good candidate for the parameterization of the selection of ASR cut-off parameters. Despite being computationally expensive, a data-driven approach for the selection process of cut-off parameters would be beneficial for the development of automatic processing pipelines.

### Limitations

Using real-world data for analyzing filtering algorithms comes with the caveat that there is no gold standard for EEG preprocessing. However, it is important to compare the results of a common preprocessing practice with newly developed automatic preprocessing algorithms as in the current study, in order to get a better understanding of the behavior of the latter. In addition, quantifiable quality features of the EEG were used to assess the effect of the cut-off parameter used in ASR. A further limitation is that the list of tasks included in this study is not exhaustive and we only used one particular type of EEG system. Thus, the results may vary for other motor tasks with different compositions of noise or different EEG amplifiers and electrodes. Nevertheless, this is the first study to investigate the effect of movement tasks, showing differences in reproducibility and dipolarity depending on the cut-off parameter used. This knowledge is important for, and may inspire, future research.

## Conclusion

Artifact subspace reconstruction appears a valuable tool for the automatic cleaning of EEG data recorded while performing motor tasks when used as a preprocessing step for an independent component analysis. We showed that the dipolarity and the reproducibility of independent components reached a combined maximum when cut-off parameters of 10 or higher were used in EEG data recorded from real participants. The number of functional ICs classified by an automatic tool remained constant, regardless of the cut-off parameter used. However, in EEG with low levels of movement induced artifacts, we observed lower combined reproducibility and dipolarity compared with more contaminated data, indicating that ASR might be less suitable for non-contaminated EEG datasets. Furthermore, ASR with cut-off parameters lower than 10 produced ICs with high dipolarity and low reproducibility.
